# The role of chemical antifouling defence in the invasion success of *Sargassum muticum*: A comparison of native and invasive brown algae

**DOI:** 10.1371/journal.pone.0189761

**Published:** 2017-12-21

**Authors:** Nicole Schwartz, Sven Rohde, Sergey Dobretsov, Shimabukuro Hiromori, Peter J. Schupp

**Affiliations:** 1 Institute for Chemistry and Biology of the Marine Environment, Carl von Ossietzky University Oldenburg, Wilhelmshaven, Germany; 2 Department of Marine Science and Fisheries and Centre of Excellence in Marine Biotechnology, Sultan Qaboos University, Muscat, Oman; 3 Center of Excellence in Marine Biotechnology, Sultan Qaboos University, Muscat, Oman; 4 National Research Institute of Fisheries and Environment of Inland Sea, Fisheries Research Agency, Hatsukaichi City, Hiroshima Prefecture, Japan; Tallinn University of Technology, ESTONIA

## Abstract

Competition and fouling defence are important traits that may facilitate invasions by non-indigenous species. The ‘novel weapons hypothesis’ (NWH) predicts that the invasive success of exotic species is closely linked to the possession of chemical defence compounds that the recipient community in the new range is not adapted to. In order to assess whether chemical defence traits contribute to invasion success, anti-bacterial, anti-quorum sensing, anti-diatom, anti-larval and anti-algal properties were investigated for the following algae: a) the invasive brown alga *Sargassum* muticum from both, its native (Japan) and invasive (Germany) range, b) the two non- or weak invasive species *Sargassum* fusiforme and *Sargassum horneri* from Japan, and c) *Fucus vesiculosus*, a native brown alga from Germany. Crude and surface extracts and lipid fractions of active extracts were tested against common fouling organisms and zygotes of a dominant competing brown alga. Extracts of the native brown alga *F*. *vesiculosus* inhibited more bacterial strains (75%) than any of the *Sargassum* spp. (17 to 29%). However, *Sargassum* spp. from Japan exhibited the strongest settlement inhibition against the diatom *Cylindrotheca closterium*, larvae of the bryozoan *Bugula neritina* and zygotes of the brown alga *F*. *vesiculosus*. Overall, extracts of *S*. *muticum* from the invasive range were less active compared to those of the native range suggesting an adaptation to lower fouling pressure and competition in the new range resulting in a shift of resource allocation from costly chemical defence to reproduction and growth. Non-invasive *Sargassum* spp. from Japan was equally defended against fouling and competitors like *S*. *muticum* from Japan indicating a necessity to include these species in European monitoring programs. The variable antifouling activity of surface and crude extracts highlights the importance to use both for an initial screening for antifouling activity.

## Introduction

Increased human travel and trade result in an unprecedented movement of organisms from all phyla across the globe. Today, invasive species represent one of the greatest threats to food security, human and animal health, and biodiversity [1]. Particular attention should be paid to marine species, since several thousand can be transported with one ship between biogeographic regions in ballast water tanks and on ship hulls alone [2, 3]. Marine algae represent a significant component of those alien or non-indigenous marine species (NIMS), comprising ∼5% of the total flora [4] and ∼10–40% of the total alien species in some regions. NIMS introduction may affect local tidal communities and whole marine ecosystems, thus becoming a global concern [5]. A better understanding of invader biology, possible ecological interactions and adaptive mechanisms is crucial for developing a targeted management and cost- and time-saving monitoring [6].

Attempting to explain the dispersal success of certain invasive species, research on chemical defence properties increasingly focuses on comparative studies of invasive and native plants [7]. The ‘‘novel weapons hypothesis” (NWH) states that some invasive species may possess novel biochemical metabolites that function as powerful weapons to which species in invasive regions have not had the opportunity to adapt [8]. The high proportion of unfamiliar metabolites of invasive species increases the chance that compounds are efficient against native species in the introduced range. Thus, the high chemical diversity and special phytochemical characteristics might be an indicator of invasion potential [7]. However, in areas of low enemy pressure, natural selection might favour genotypes with reduced resource allocation to defence, but improved competitive abilities by maximised vegetative growth and reproduction as postulated by the ‘Evolution of increased competitive ability hypothesis’ (EICA; [9]). Similarly, the reduced allocation to costly resistance and the concurrent reallocation of resources to reproduction and growth might occur through phenotypic plasticity, which is known as the ‘Plastic Increase in Competitive Ability Hypothesis’ (PICA; [10]). Development of both hypotheses was motivated by the ‘the dilemma of plants: to grow or defend’ [11], which assumes a costly production of secondary metabolites and a consequential trade-off between production of defence metabolites and growth or reproduction.

Novel metabolites might protect introduced algae from being overgrown, a process known as epibiosis or biofouling [12], thus, providing them with a competitive advantage and aiding their dispersal compared to native co-occurring algae. Biofouling on algal surfaces might have different adverse effects for the algal host including reduced photosynthesis, gas exchange and nutrient uptake, as well as diseases and tissue necrosis induced by bacteria and fungi [12, 13]. In order to protect their surfaces from fouling organisms, macroalgae can produce a wide variety of defence metabolites [14, 15]. This chemical antifouling defence might either reduce epibiosis directly or control the composition of the microfouling community on their surfaces, which can itself excrete antifouling metabolites to prevent further macrofouling [16, 17].

Some algal metabolites are known to inhibit bacterial quorum sensing (QS), a densitiy dependent microbial cell-to-cell communication [17, 18]. Inhibition of bacterial QS has been reported as a strategy for the control of biofilm formation used by several marine organisms [19].

The invasive alga *Sargassum muticum* (Yendo) Fensholt 1955 is a well-defended member of the Sargassaceae family and a rich source of phenolic compounds, which are known to possess, amongst others, deterrent characteristics against e.g. herbivores and larval settlement [20, 21]. Native to Japan, it has spread widely along the European Atlantic coasts since its unintentional introduction with oysters in the early 1970s [22]. It reached the German part of the North Sea in 1988 [23] and affected and reduced the marine benthic algal community by competing with local co-occurring species and shaping the local ecosystem [24, 25]. The effective chemical defence of *S*. *muticum* might contribute to its invasiveness and the comparison of this strong invader with native, closely related species from Japan might provide an insight into species traits contributing to invasion success.

In the current study, we compared antifouling defences and chemical metabolites of invasive *S*. *muticum*, non-invasive *Sargassum fusiforme* (Harvey) Setchell and *Sargassum horneri* (Turner) C. Agardh, an algal species that has recently been detected outside its native range from Japan and the local dominant North Sea brown alga *Fucus vesiculosus* Linnaeus 1753. In order to detect adaptations in chemical defence properties after the invasion process we additionally compared extracts of an invasive *S*. *muticum* population from the German North Sea with a native allopatric population from Japan. Antifouling and anti-settlement assays were conducted with dominant fouling organisms using algal surface and crude extracts in different concentrations to test the following hypotheses:

1. Algal surface extracts have stronger antifouling activity compared to crude extracts.
2. *S*. *muticum* is better defended against fouling compared to the local co-occurring brown alga *F*. *vesiculosus* as predicted by the NWH.
3. *S*. *muticum* from Japan is better defended against local North Sea foulers and competitors compared to *S*. *muticum* from the North Sea.
4. *S*. *muticum* is better defended against fouling and competing algae than its native relatives explaining its invasive success in the North Sea.

## Materials and methods

### Collection of algae

Thalli of three Japanese *Sargassum* species, *S*. *fusiforme*, *S*. *horneri* and the native *S*. *muticum*, hereafter *S*. *muticum* (nat.), were collected by Scuba diving in Oshima, Japan (33°55′04.4"N 132°27′42.7"E—33°56′26.3"N 132°24′06.0"E) during January and March 2013 at a depth of 1–3 m. The invasive population of *S*. *muticum*, hereafter *S*. *muticum* (inv.), was collected in Germany in April 2012 for antibacterial assays by hand at low tide in the shallow subtidal zone of an oyster reef in the Wadden Sea of Lower Saxony, Germany (53°38.415’N, 6°56.359’E) and again in June 2014 for settlement assays in the sheltered rocky intertidal zone of Heligoland, Germany (54° 11′ 10.38″ N, 7° 52′ 18.87″ E). *Fucus vesiculosus* was collected by hand from Nassau harbour in Wilhelmshaven, Germany (53°30′54.7"N 8°08′57.8"E) in April 2012 for anti-bacterial assays and again in July 2015 for anti-settlement assays. After collection, all algal specimens were immediately transferred to the laboratory in insulated coolers filled with seawater. At the laboratory, algae were gently washed with sterile seawater and cleaned of associated microflora. The wet weight (± 0.01 g) was measured by a balance. The volume of each specimen was determined by water displacement in a graduated cylinder. One part of fresh algal material from Japan was wrapped in wetted cloth and sent cooled to Germany by express carrier for surface extraction. The other part was freeze-dried and subsequently sent to Germany for crude extraction. One part of *S*. *muticum* and *F*. *vesiculosus* from Germany were used for surface extraction and another part was freeze-dried, weighed again, ground into fine powder to increase extraction efficiency and extracted.

### Preparation of surface extracts

Surface-associated metabolites from fresh algal material from Japan and Germany were obtained by using a modification of the ‘dipping’ technique [26]. Individual thalli were dipped under continuous stirring in hexane/methanol (1:1, v/v) for 10 sec [27]. The combined surface extracts of all individuals of one algal species were filtered through Watman No1 filter (Sigma-Aldrich, Germany) and solvents were removed by rotary evaporation (Büchi, Switzerland). Obtained extracts were dried in a centrifugal vacuum concentrator (Christ RVC 2–25). Extracts were weighed and stored in a freezer at– 20°C until used in bioassays (Table 1).

**Table 1:** Biomass of tested brown algae and their specific extract yield.

Algal species	Sampling location	Crude extract	Crude extract	Crude extract	Crude extract	Surface extract	Surface extract	Surface extract	Surface extract
		Dw_alga_ [g]	Vol_alga_ [ml]	Extract [g]	Nat. conc. [mg ml^-1^]	Ww_alga_ [g]	Vol_alga_ [ml]	Extract [g]	Nat. conc. [mg ml^-1^]
*S*.*f*.	Seto Inland Sea	55	362,1	8	22	494,4	427,8	3,3	7,6
*S*.*h*.	Seto Inland Sea	45	397,8	9,8	24	526,9	455,9	9,8	21,6
*S*.*m*.	Seto Inland Sea	60	474,8	13,8	29	581,5	672	8,7	12,9
F.v.	North Sea	60	306,7	10,9	35,4	375,5	660	2,6	3,9
S.m.	North Sea	104,4	645,9	19,5	30,2	1203,4	1072	8,5	7,9

Dry- and wet weight (dw and ww), the respective volume (vol.) of extracted *S*. *fusiforme* (*S*.*f*.), *S*. *horneri* (*S*.*h*.), *S*. *muticum* (*S*.*m*.) and *Fucus vesiculosus* (*F*.*v*.) and the yielded crude and surface extract with the specific natural tissue-concentration (nat. conc.).

### Preparation of crude extracts

Freeze-dried algal powder of each species was extracted three times with ethyl acetate/methanol (EtOAc/MeOH, AppliChem GmbH) (1:1) and finally with 100% MeOH (AppliChem GmbH). For each gram of algal dry weight 20 ml of solvent solution was used. Each extract was filtered through Watman No1 filter and remaining solvents were removed by rotary evaporation. Obtained extracts from the same algal species were combined, weighed and stored in a freezer at– 20°C until used in bioassays (Table 1).

Before the experiments extracts were re-dissolved to desired concentration using MeOH or EtOAc/MeOH (1:1).

### Antibacterial assay

#### Isolation of marine bacteria

Marine bacteria were obtained by surface swabs with sterile cotton buds from 7 d old biofilms developed on glass slides exposed to the sea water in Banyuls, France (42°28′52.9"N 3°08′15.0") (strain 1), and Wilhelmshaven, Germany (53°30′54.5"N 8°08′57.8"E) (strains 2–4). The swabs were placed onto Petri dishes containing marine agar (1.5% agar, 37.4 g l^-1^ Difco Marine Broth 2216, filtered de-ionized water) and incubated for 24 h at room temperature. Distinguishable colonies were selected by colony morphology and purified by repeated single colony subculture on the same media. Two bacterial strains (strain 5 and 6) causing algal thalli bleaching were obtained from the Centre of Marine Bio-Innovation, University of New South Wales, Sydney, Australia [28, 29]. Both were isolated from the epiphytic bacterial community of the red alga *Delisea pulchra*. *Chromobacterium violaceum* CV026 (strain 7) was obtained from the culture collection of Sultan Qaboos University (SQUMSF-026) and grown in LB medium (Luria Bertani Broth, BD, USA).

#### Identification of bacterial strains

For bacterial DNA extraction, a single pure colony of each culture was frozen with 50 μL PCR water and defrosted 3 times. Polymerase chain reaction (PCR) amplification was performed using a total volume of 25 μL containing 10x PCR buffer (Qiagen, Gaithersburg, MD), 250 μM of each desoxynucleotide, BSA (30 mg/mL), 100 mM MgCl_2_, 1.25 U Taq DNA polymerase (Qiagen, Gaithersburg, MD), 1 μl bacterial DNA, and the bacterial primers 27F (GAG TTT GAT CCT GGC TCA) and 1492R (TAC GGY TAC CTT GTT ACG ACT T) [30]. The PCR cycling conditions were: 95°C for 5 min; 30 cycles at 95°C for 1 min, 55°C for 1 min, 72°C for 90 s; followed by 72°C for 10 min. PCR products were separated on 1% agarose gels stained with ethidium bromide and visualized under UV illumination. DNA was purified to remove excess primers and unused material using the QIAquick PCR Purification Kit (Qiagen, Gaithersburg, MD) following the manufacturer’s protocol. PCR products were sequenced by GATC Biotech AG. Sequence chromatograms were viewed and edited using BioEdit sequence alignment editor Version 7.2.5 [31]. High quality sequences (defined as >600 bp) were compared to the National Center for Biotechnology Information (NCBI) database (http://blast.ncbi.nlm.nih.gov) by using BLAST (Table 2).

**Table 2:** Blast identification and percentage similarity of bacterial 16S rDNA sequences.

Strain No.	Strain Source	Gram staining	Accession No.	Blast Identification (% of similarity)
1	Banyuls, France	-	NR_025195.1	*Shewanella fidelis* (99%)
			AF500079	*Shewanella japonica* (99%)
2	Wilhelmshaven, Germany	+	FR821137.1	*Bacillus* sp. (99%)
			AB362273.1	*Bacillus hwajinpoensis* (99%)
3	Whv, Germany	-	KJ917337.1	*Pseudoalteromonas lipolytica* (97%)
			KF6552.1	*Pseudoalteromonas* sp. (97%)
4	Whv, Germany	-	KF933699.1	*Halomonas sp*. (98%)
			JQ924079.1	*Actinomycetales bacterium* (98%)
			AM110924.1	*Bacillus subtilis* (98%)
			KF933711.1	*Cobetia marina* (98%)
5	Sydney, Australia	-		*Phaeobacter gallaeciensis* LSS9
6		-		*Nautella sp*. R11
7	Muscat, Oman	-		*Chromobacterium violaceum* CV026

Strains 1–4: isolated marine bacterial strains; strains 5–7: obtained bacterial strains.

### Antibacterial activity

#### Construction of bacterial growth curve

Antibacterial activity of algal extracts was assessed by measuring bacterial growth of the isolates listed in Table 2. All bacterial cultures were grown overnight in liquid Media at 20°C. Before each bioassay bacteria were diluted with fresh media to adjust the optical density (OD) at 600 nm to OD_600_ = 0.123 ± 0.003. To test the natural inhibitory effect of algal extracts, crude and surface extracts were re-dissolved to a natural concentration and subsequently added to 96-well plates (Nunc 96 MicroWell^™^ polystyrene Plates—MaxiSorp^™^). After complete evaporation of the solvent, 40 μl of liquid medium was added to every well to provide enough nutrients for bacterial growth. Plates were left incubating for 2 h at 20°C on a shaker with 120 rpm. This incubation time was required to pre-dissolve the algal extracts. Subsequently, 40 μl of bacteria containing media was added. The antibiotics gentamicin and, in the case of strain 1, penicillin (100μg/ml; Roth, Germany) served as positive controls, whereas the solvent EtOAc/MeOH was used as a negative control. Wells without solvent were used as additional controls to assure that solvent itself had no effect on bacterial growth. Each treatment was replicated six times. Bacterial OD was measured with a microplate reader (Thermo Scientific Multiscan FC, Type: 357). A turbidimetric growth curve was generated by measuring the optical density at 600 nm after 0, 1, 2, 4, 6, 10, 15, 24, 30, 36 and 48 h from the start of the experiment and the specific slope of each growth curve was determined. All measurements were done in triplicates. In order to assess the intensity of bacterial growth inhibition quantitatively, the log response ratio (LogRR) was calculated as LogRR = log (T/C), where T is the mean slope of bacterial growth with the presence of either algal extracts or antibiotics, and C is the mean slope of bacterial growth with the solvent control. A positive value of LogRR indicates stimulation and a negative value indicates inhibition of bacterial growth [32].

#### Anti-quorum sensing (QS) assay and separation of compounds

QS inhibitory activity of *Sargassum* spp. extracts was tested using the bacterial reporter strain *C*. *violaceum* CV017 [33]. *C*. *violaceum* CV017 is commonly used for screening anti-QS activity due to the production of the purple pigment violacein in response to short side-chain AHL’s [34; 35]. The reporter strain was obtained from the culture collection of Sultan Qaboos University (SQUMSF-017) and grown in LB medium at 30°C over night.

*Sargassum* spp. crude and surface extracts were diluted with EtOAc:MeOH (1:1) to prepare 1-, 2-, 3-, 4- and 6-times natural concentrations. 15 μl of each extract at each concentration and the control EtOAc:MeOH (1:1) were applied onto sterile cellulose paper discs (diameter = 6 mm). The reporter strain was inoculated onto the agar plates (LB Medium containing 1.5% agar) and dried discs with either extract or the solvent (EtOAc/MeOH) control and additional empty discs were placed upside down onto the agar. After overnight incubation at 30°C, a visible purple bacterial lawn had developed. Observed transparent zones around the disks indicated QS inhibition. The zones were measured to the nearest 0.2 mm with a ruler and the minimum inhibitory concentration (MIC) was determined for each extract. In order to exclude the possibility of antibacterial activity against the reporter strain, antibacterial experiments with *C*. *violaceum* CV026 were conducted (see above).

Extracts exhibiting QS-inhibitory properties were further separated by thin layer chromatography (TLC) to detect the polarity of active compounds [36, 37]. Re-dissolved extracts were applied to C18 reversed-phase TLC plates (200 μm layer; Baker) with a mixture of DCM: EtOAc: hydrochloric acid 10% solution (18:1:1) as mobile phase. After development of the plates in a TLC chamber, plates were dried and rimmed with a 2 mm high frame, designed to hold a uniform thin bacterial layer, and overlaid with agar containing the indicator bacteria CV017. Therefore, 2 ml of an overnight grown bacterial culture was added to 10 ml of the same medium containing 1.5% agar maintained at 45°C. The culture was mixed thoroughly and immediately spread over the TLC plates and incubated at 30°C for 12 h. The solvent served as controls. Each extract at each concentration was tested in triplicate.

### Antidiatom assay

Natural concentrations of all algal extracts were tested against the benthic diatom, *Cylindrotheca closterium* ([38]; CCAP 1017/8), a well known fouling species [39, 40], obtained from the CCAP (Culture Collection of Algae and Protozoa, SAMS Research Services Ltd., Scottish Marine Institute, Dunbeg, Argyll, UK). To determine the minimum inhibitory concentration (MIC) of algal extracts series of four dilutions (1, 0.5, 0.1, 0.05, 0.01 times of natural tissue-concentration) were tested.

Prior to the experiments diatom cultures were grown under controlled conditions at 17.8°C with 16 ± 0.5 μmol photons m^-2^ s^-1^ cool-white fluorescent light, with a 12:12 h light-dark cycle in F/2 medium [41]. Extracts and controls were applied at different concentrations to 24 well plates (TPP, polystyrene, Sigma-Aldrich). Empty wells and MeOH served as controls. After complete evaporation of solvents, 1 ml of diatom culture (10.3 ± 0.75 cells x 10^4^ ml^-1^) was added and allowed to grow and attach for 7 days at culture conditions (see above). Six replicates were used for each extract and concentration. At the end of the experiment, diatom densities were determined using a microscope (Leica microsystems CMS GmbH; magnification 400 x). Diatoms were counted in 10 randomly selected fields of view (0.25 mm^2^) and diatom densities in treatments were compared with the controls to assess possible growth inhibition of algal extracts. Changes in diatom morphology were additionally recorded.

### Bryozoan larval settlement bioassay

Adult bryozoan colonies of *Bugula neritina* were collected from submerged ship hulls and ropes hanging on the pontoons of the Marina Bander Al Rowdah, Muscat, Oman (23° 34′ 55″ N 58° 36′ 27″ E). In the laboratory, bryozoans were stored in dark, aerated buckets (4 l) filled with filtered seawater (pore size = 0.45 μm) for the next 24 h. Exposure to sunlight induced the release of larvae which were collected with a pipette (for details see [42]) and immediately used in settlement assays. The assay was conducted using natural concentrated and 0.2-times natural concentrated extracts of *Sargassum* spp. Extracts were added to 24 well plates (Corning^®^ Costar^®^, USA), which were kept under the fume hood until the solvents were completely evaporated. MeOH and wells without extracts served as controls. Subsequently, 1 ml of filtered (pore size = 0.45 μm) seawater containing 10–20 bryozoan larvae was added to each well. Numbers of settled, swimming and dead larvae in each well were counted after 1, 3, 6 and 24 h using a stereo microscope (Zeiss, Axiostar plus). Larvae were scored as percent settled following attachment and metamorphosis, which is marked by eversion of the metasomal internal sac [43]. The assays were conducted with 10 replicates.

### Anti-algal activity

#### Collection of *F*. *vesiculosus* zygotes

*F*. *vesiculosus* receptacles were collected from Nassau harbour, Wilhelmshaven (53.5144° N, 8.1450° E) in May 2015, rinsed with fresh water and stored in the dark at 7°C for five days. Fronds were then induced to release gametes in filtered seawater (FSW; 0.14 mm) under strong fluorescent lights (52.21 μmol ± 0.5 μmol photons m^-2^ s^-1^). Released eggs were removed from the bottom and gently mixed with the sperm suspension on a magnetic stirrer for additional 15 min to ensure fertilization.

#### Zygote settlement and development assay

*Sargassum* spp. extracts and the controls were applied individually to 12 well plates (TPP, polystyrene, Sigma-Aldrich) at natural, 0.5- and 0.1-times natural concentrations. Each extract concentration and solvent control was replicated six times. After complete evaporation of the solvent, 1 ml of continuously stirred zygote suspension was added and directly counted to determine the start concentration for each well. Plates were stored at room temperature under fluorescent light (52.21 μmol ± 0.5 μmol photons m-2 s-1) with a 10:14 h and a 14:10 h light-dark cycle for the December and the April assay, respectively. After 24, 48, 68, 164, and 260 h wells were gently rinsed with filtered seawater to remove not-attached zygotes. 24 h was discovered as a sufficient time for *Fucus* zygotes to settle in a previous experiment [44]. Remaining zygotes were counted under a stereo microscope (Leica microsystems CMS GmbH; magnification 2.5 x 10) in five fields of view (1.25 cm^2^) and rhizoid formation was recorded. The MIC of each extract was calculated.

### Quantification of polyphenols

The total phenolic content of all species used in the feeding assays was determined with a microplate-adapted Folin–Cioalteu assay following the procedure described in [45]. Phloroglucinol (1,3,5-trihydroxybenzene, Sigma-Aldrich, Germany) was used as a standard and a calibration curve was generated with concentrations of 0, 6.25, 12.5, 25, 50 and 100 μg mL^-1^. Total phenolic contents (TPCs) were expressed as percentages of phenolic compounds per algal dry weight. The Folin-Ciocalteu method quantifies nonphenolic hydroxylated aromatic compounds as well, but since these interfering substances make up <5% of the total reactive compounds, they were neglected [46]. In the following, the term ‘phlorotannins’ is used for total phenolics, since brown algae are not known to contain other polyphenols [47].

### Statistics

Statistical calculations were performed using the SPSS IBM Statistics version 23, Illinois, USA software. Normality and homogeneity of variances were determined using Kolmogorov–Smirnov test and Levene’s tests respectively. When no homoscedasticity was achieved, the non-parametric Kruskal-Wallis test with subsequent multiple comparisons was applied. In all cases the significance level was set at α < 0.05.

A one-way ANOVA was used to assess effects of algal extracts on bacterial growth and anti-settlement activity of *Sargassum* extracts on *Fucus* zygotes. Differences between extracts and controls were determined with subsequent Tukey post-hoc tests.

Diatom- and bryozoan larval settlement did not always meet the assumptions of ANOVA. Therefore, the non-parametric Kruskal-Wallis test followed by Dunn’s multiple comparison test was applied [48].

## Results

### Antibacterial activity

#### Growth inhibition

**Fig 1:**
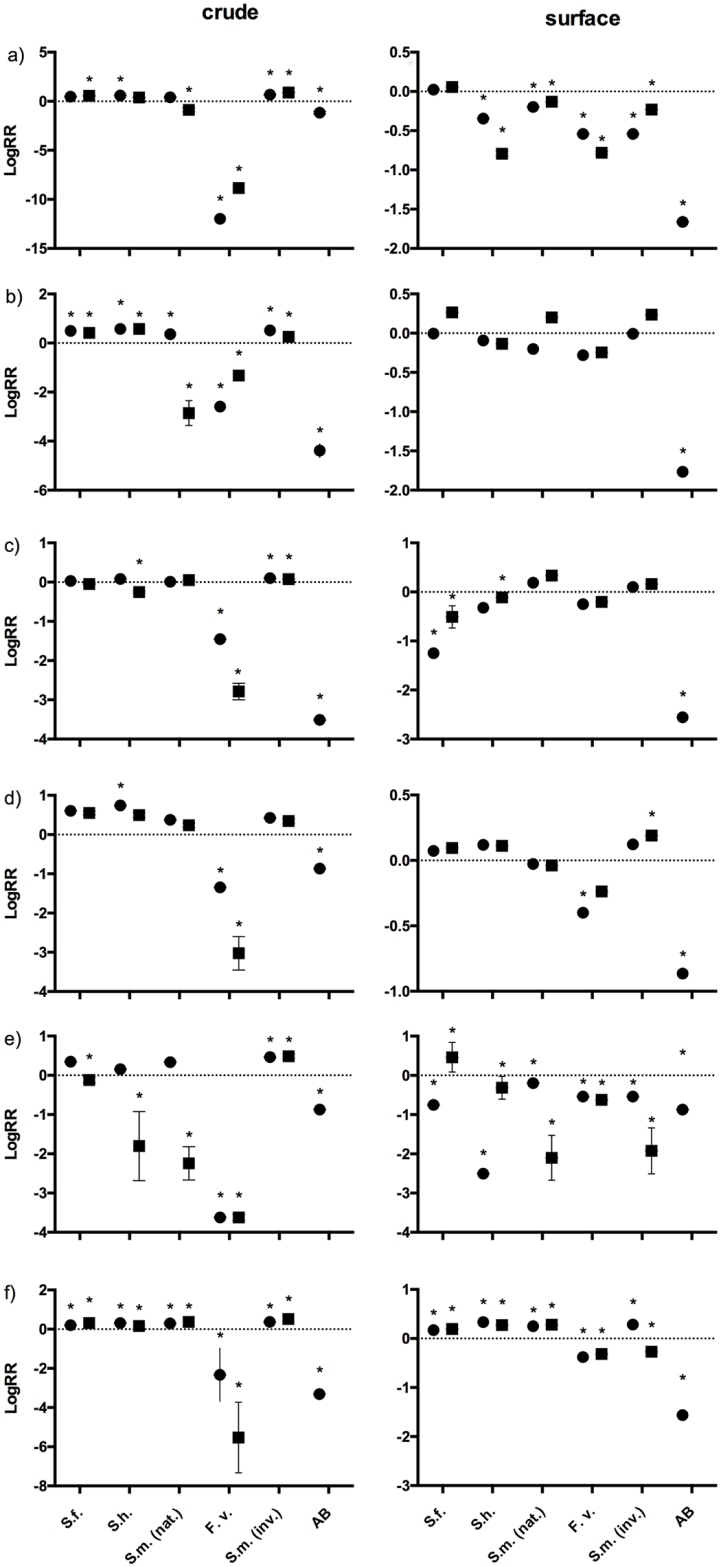
Bacterial growth inhibition. Log response ratios (LogRR) of bacterial growth of strains 1–6 (a-f) in response to different concentrations of crude and surface extracts of *Sargassum fusiforme* (*S*.*f*.), *Sargassum horneri* (*S*.*h*.), native (nat.) and invasive (inv.) *Sargassum muticum* (*S*.*m*.), *Fucus vesiculosus* (*F*.*v*.) and the antibiotic positive control (AB; gentamicin or penicilin; 100 μg/ml). Circle–natural concentrations, square– 2-times natural concentrated extracts. Asterisks indicate significant differences between treatments and solvent control (ANOVA, Tukey post-hoc test p < 0.05).

The antibacterial assays revealed significant stimulatory (27.5%) or inhibitory activity (34.17%) of tested extracts (Fig 1). Overall, the crude extract of invasive *S*. *muticum* significantly increased bacterial growth rates by 83%. In opposite, *F*. *vesiculosus* crude extracts exhibited the strongest bacterial growth inhibition and they were effective against all test strains. Although all *Sargassum* surface extracts showed inhibitory activities, extracts were not effective against all tested bacterial strains (Fig 1). In some cases, lower concentrations had a stronger antibacterial effect than the higher concentrations of extracts. For example, *S*. *horneri* natural concentrated crude extract reduced bacterial growth significantly (ANOVA, Tukey post-hoc test p < 0.05) whereas the two times concentrated extract had no effect. The same pattern was observed with the crude extract of native *S*. *muticum* (Fig 1). Antibacterial activity was slightly higher with surface extracts (38%) than with crude ones (30%), while crude extracts exhibited the 2.7-times higher stimulatory activity compared to surface extracts.

#### Anti-QS activity

Crude and surface extracts of *S*. *fusiforme* (at 88.08 and 30.40 mg ml^-1^, resp.) as well as crude extracts of native *S*. *muticum* (115.84 mg ml^-1^) inhibited QS of the reporter CV017 at 4-times natural concentrations (Table 3). However, *S*. *muticum* (nat.) extracts at the natural concentration (29 mg ml^-1)^ reduced the growth of *C*.*violaceum* CV026. Agar overlays of TLCs revealed that QS inhibitors were unpolar compounds (S1 Fig).

**Table 3:** Anti-QS and growth inhibitiory activity of *Chromobacterium violaceum* by algal extracts.

Species	Extract	MIC of QS inhibition	MIC of QS inhibition	MIC of QS toxicity	MIC of QS toxicity
Species	Extract	x N	mg ml^-1^	x N	mg ml^-1^
*S*.*f*	surface	4	30.40	-	-
*S*.*f*.	crude	4	88.08	-	-
*S*.*h*.	surface	-	-	-	-
*S*.*h*.	crude	-	-	-	-
*S*.*m*. (nat.)	surface	-	-	-	-
*S*.*m*. (nat.)	crude	4	115.84	1	29
*F*.*v*.	surface	-	-	-	-
*F*.*v*.	crude	-	-	-	-
*S*.*m*. (*inv*.)	surface	-	-	-	-
*S*.*m*. (*inv*.)	crude	-	-	-	-

Comparison of extracts of native and invasive *S*. *muticum* (*S*.*m*.), *S*. *fusiforme* (*S*.*f*.), and *S*. *horneri* (*S*.*h*.) and *Fucus vesiculosus* (*F*.*v*.) in quorum sensing (QS) inhibition bioassay using the reporter *Chromobacterium violaceum* CV017 and the toxicity test using the strain *Chromobacterium violaceum* CV026. Minimum inhibitory concentration (MIC) is expressed as times natural tissue level concentration (x N) and mg ml-1. (-) = no effect. Data are the means of three replicates. There were no differences between replicates.

### Settlement inhibition

#### Antidiatom activity

**Fig 2:**
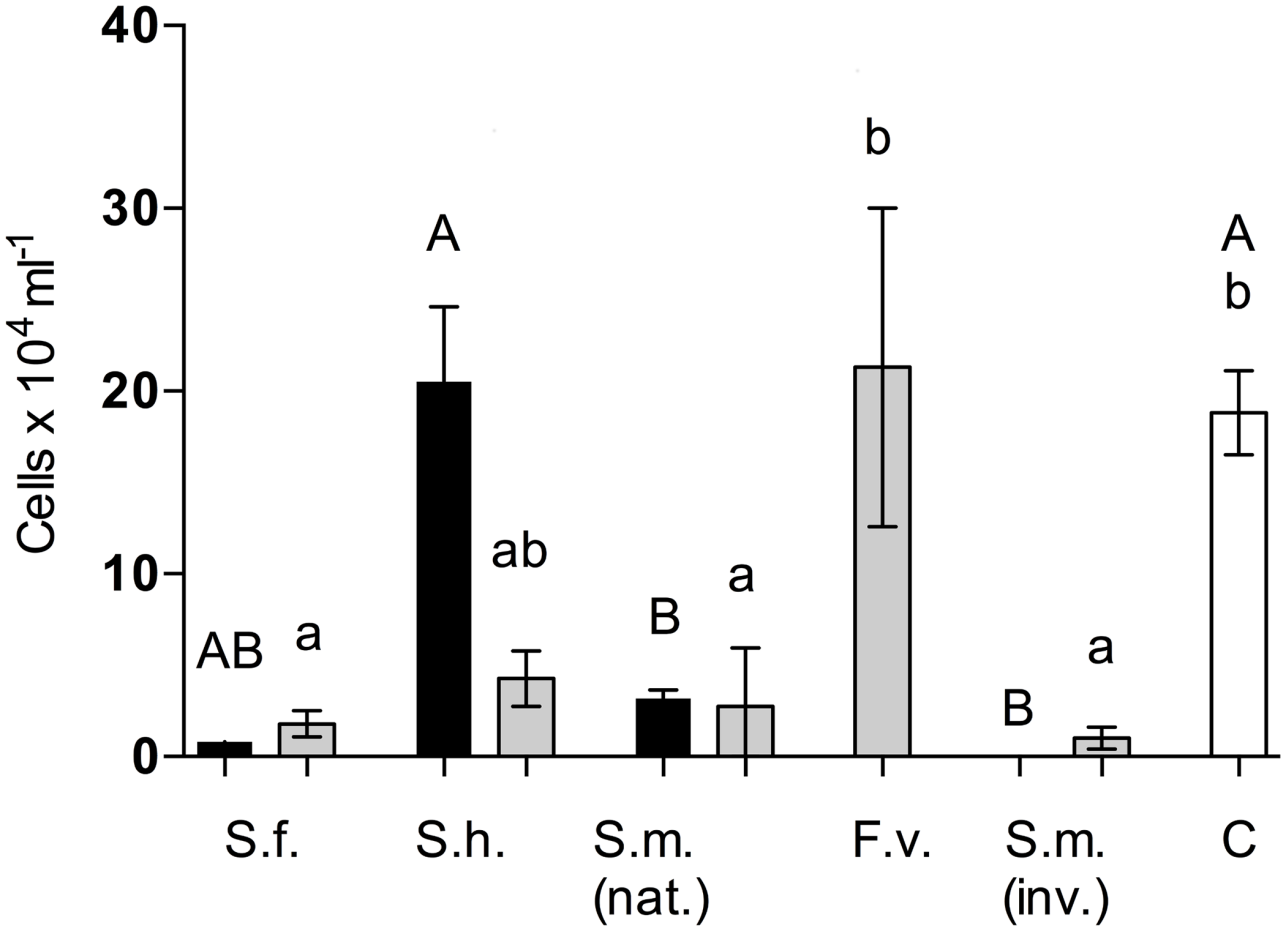
Antidiatom activity of algal extracts. Effect of crude (black) and surface (grey) extracts of *S*. *fusiforme* (*S*.*f*.), *S*. *horneri* (*S*.*h*.), native and invasive *S*. *muticum* (*S*.*m*. (nat.) and (inv.)), and *F*. *vesiculosus* (*F*.*v*.) on cell densities (mean ± SD, n = 6) of the diatom *C*. *closterium* after 7 days. Extracts were tested at the natural concentrations. Methanol was used as the control (C). Significant differences (p < 0.05) are indicated by different letters (upper- and lower case letters for crude and surface extracts, respectively) above the bars (according to Kruskal–Wallis test with the Dunn′s multiple post-hoc comparison test).

Because of the dark colour of *F*. *vesiculosus* crude extracts it was not possible to quantify its effect on diatom densities. All other tested crude and surface extracts except crude extract of *S*. *horneri* and surface extracts of *F*. *vesiculosus* reduced more than 50% of the densities of diatoms compared to the solvent controls. Crude and surface extracts of most *Sargassum* extracts reduced densities of *Cylindrotheca closterium* significantly compared to the control (p < 0.05, Dunn′s test, Fig 2). Although the invasive *S*. *muticum* extracts had the strongest effect compared to the native ones, the difference was not statistically significant (p < 0.05, Dunn′s test, Fig 2).

Besides affecting diatom density there were pronounced differences in morphology of *C*. *closterium* cells exposed to the different treatments. Diatom cells growing in the presence of crude extracts of *S*. *horneri* were pale in colour, their frustules were broken and released chloroplasts covered the bottom of the wells. On the other hand, in the control treatment *C*. *closterium* cells were healthy with only few broken cells and free chloroplasts. Diatoms exposed to *F*. *vesiculosus* crude extracts looked healthy and the cells were intact (S2 Fig).

**Fig 3:**
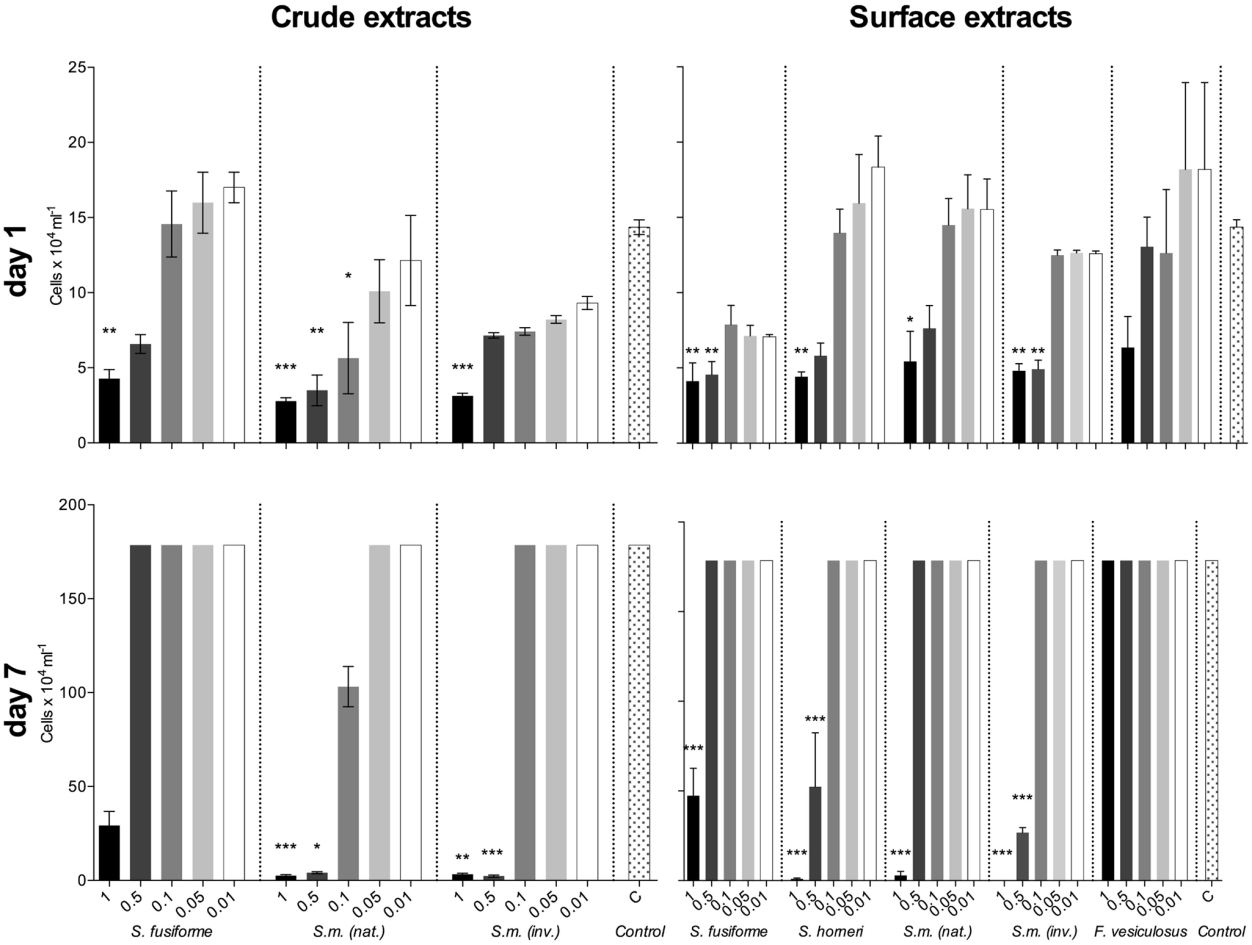
Growth inhibitory activity of algal extracts on *Cylindrotheca closterium*. Effect of different concentrations (1-, 0.5-, 0.1-, 0.05-, and 0.01-times natural concentration) of crude and surface extracts of *S*. *fusiforme*, the native (nat) and invasive (inv) *S*. *muticum* (*S*. *m*.) and surface extracts of *S*. *horneri* and *F*. *vesiculosus* on the density of the diatom *Cylindrotheca closterium* after 1 and 7 days. The data are the mean + SD, n = 6. Colour gradient from black to white represents different concentrations of extracts. Significant density differences compared to controls are indicated by asterisks above the bars (* p ≤ 0.05, ** p ≤ 0.01, ***p ≤ 0.001, Kruskal–Wallis test).

The second assay that used a dilution series of active crude and surface extracts showed that after one day, crude extracts of the native *S*. *muticum* had the strongest growth inhibition (p < 0.05, Fig 3) and a MIC of 0.1-times natural concentration. Anti-diatom effects of diluted crude extracts of the native *S*. *muticum* and surface extracts of *S*. *fusiforme* decreased with increasing assay duration (Fig 3). After 7 days, 0.5-times natural concentrated extracts of native and invasive *S*. *muticum* and surface extracts of *S*. *horneri* significantly reduced diatom growth compared to the control (p < 0.05, Fig 3). In these treatments broken frustules and released chloroplasts covering the bottom of these wells were observed. *F*. *vesiculosus* surface extracts at the natural concentration had a slight but not significant inhibitory activity.

#### Antilarval activity

Natural concentrations of crude and surface extracts of nearly all *Sargassum* spp. exhibited pronounced toxicity and killed more than 80% of bryozoan larvae compared to the controls (data not shown). Only larvae growing with natural concentrated *S*. *fusiforme* surface extracts survived and settled at rates of about 50%.

**Fig 4:**
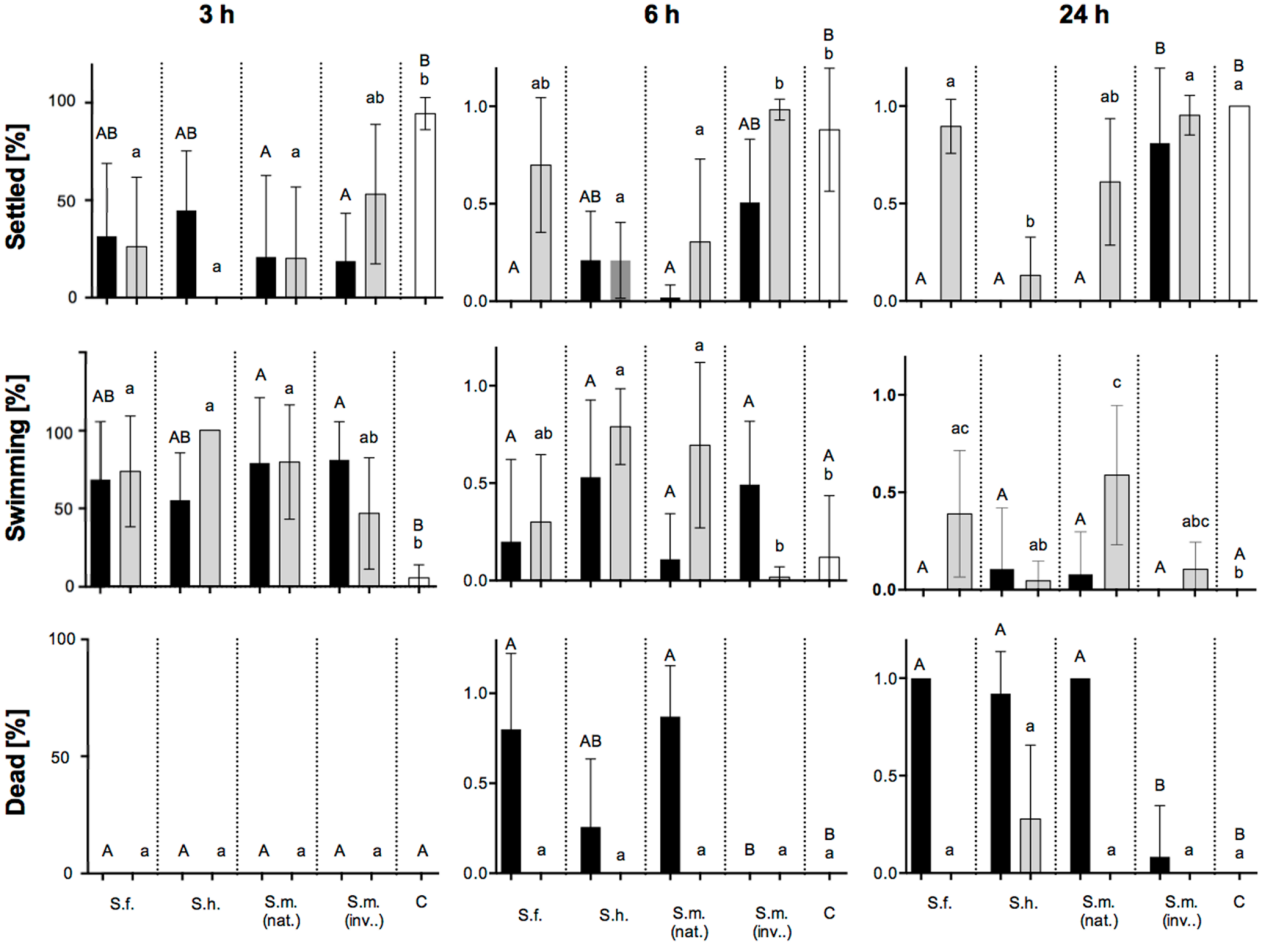
Anti-larval activity of algal extracts. Percentage of settled, swimming and dead bryozoan larvae of *B*. *neritina*. Larvae were exposed to 0.2-times natural concentrated crude- (black) and surface extracts (grey) of *S*. *fusiforme* (S.f.), *S*. *horneri* (S.h.) the native and invasive *S*. *muticum* (S.m. (nat.) and S.m. (inv.)) and solvent controls (C). Larvae were counted 3, 6, and 24 h after the start of the experiment. The data are the mean + SD, n = 10. Significant differences (p < 0.05) of crude (capital letters) and surface extracts (lowercase letters) compared to the controls are indicated by different letters above the bars (Kruskal–Wallis test with Dunn′s multiple comparisons post hoc test).

Therefore, the effects of 0.2-times natural concentrated crude and surface extracts were tested (Fig 4). After three hours 77% of all larvae settled and metamorphosed in the controls. In contrast, all extracts effected the settlement of larvae. Larval settlement was the highest (53%) in the presence of surface extracts of invasive *S*. *muticum* (inv.) and the lowest (0%) in the presence of surface extracts of *S*. *horneri*. After six hours exposure to 0.2-times natural concentrated crude extracts, larval mortality was the highest in the presence of *S*. *muticum* (nat.) and the lowest in the case of *S*. *horneri* extracts (26%). However, surface extracts of the invasive *S*. *muticum* and S. *fusiforme* had the highest proportion of settled larvae (98% and 70%, correspondingly). Overall, crude extracts of *S*. *muticum* (nat.) and *S*. *fusiforme* exhibited the highest mortality of *Bugula* larvae only after six hours of exposure. Surface extracts of all *Sargassum* spp. had the lowest anti-larval properties compared to the respective crude extracts of the same species. Only surface extracts of *S*. *muticum* (nat.) and *S*. *horneri* showed significant settlement deterrence compared to the control (Fig 4, p < 0.05, Dunn′s test).

#### Effects on *Fucus* zygote development

**Fig 5:**
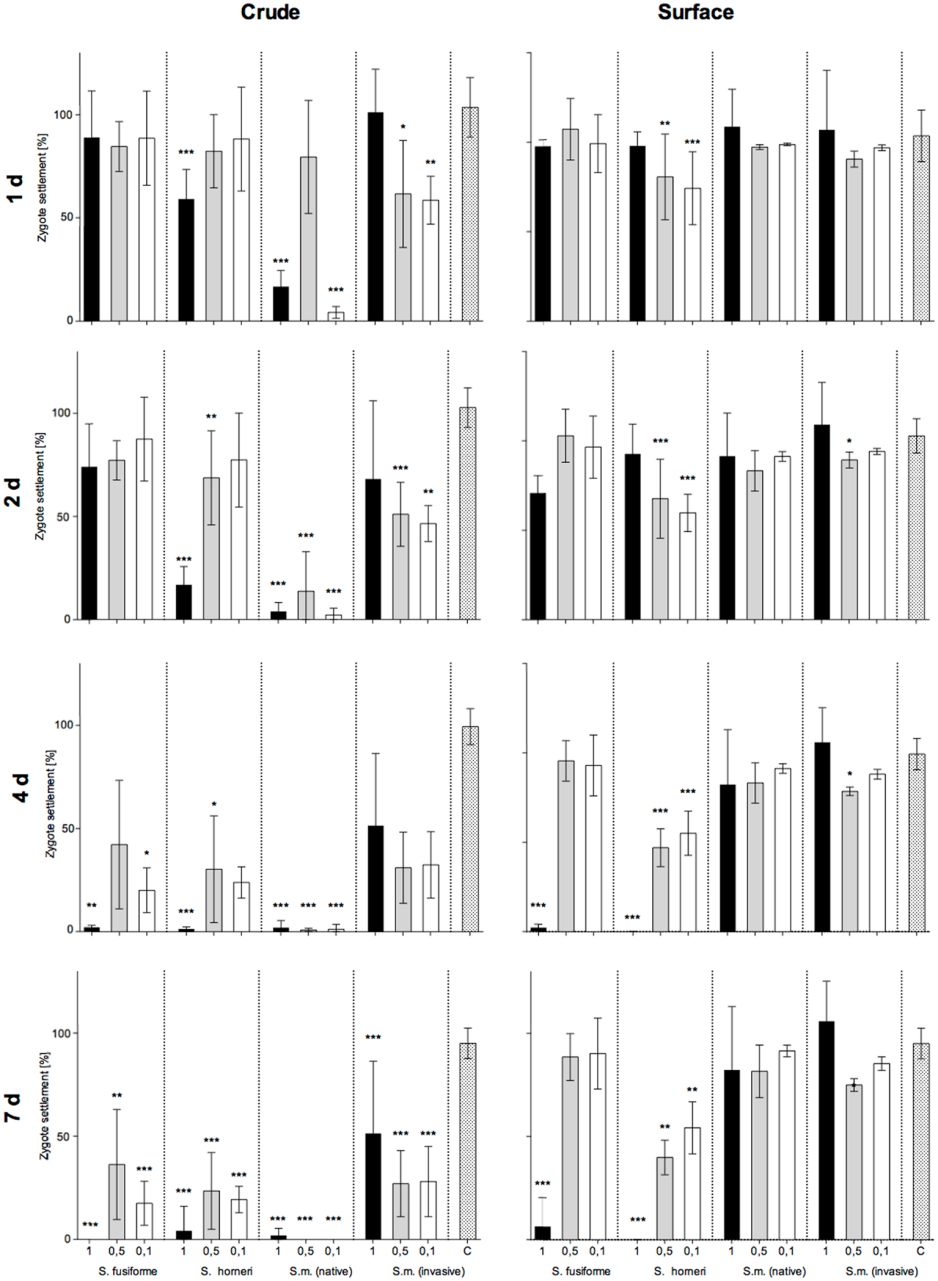
Anti-zygote activity of algal extracts. Effect of different concentrations (1-, 0.5-, 0.1-times natural concentrations) of crude and surface extracts of *S*. *fusiforme*, *S*. *horneri*, the native and invasive *S*. *muticum* (S.m.) and solvent controls (C) on *F*. *vesiculosus* zygote settlement. Data are the mean ± SD, n = 6. Settlement rates were scored 1, 2, 4 and 7 days after the start. Significant settlement differences compared to controls are indicated by asterisks above the bars (* p ≤ 0.05, ** p ≤ 0.01, ***p ≤ 0.001, ANOVA).

*Fucus* zygote development was observed over seven days with different concentrations of crude and surface extracts *of Sargassum* spp. There were visible morphological differences in zygote development depending on treatments. Rhizoid formation was prevented or slowed down with all *Sargassum* crude extracts, regardless of the concentration. After two days, all (100%) zygotes were still growing in the control, whereas < 5% of alive zygotes remained in the presence of native *S*. *muticum* crude extracts, even at the lowest tested concentration (Fig 5).

Crude extracts of native *S*. *muticum* had overall the strongest inhibitory effect at 0.5- and 0.1-times natural concentrations. On the contrary, surface extracts of the native *Sargassum* species exhibited no significant settlement inhibition, except for *S*. *horneri*, which inhibited zygote settlement completely (Fig 5). Interestingly the lower concentrations of extracts of certain *Sargassum* species had a stronger effect than the higher concentration of the same species. For example crude extract of *S*. *muticum* (nat.) at 0.5-times natural concentration had about 80% settled zygotes while 0.1-times had about 5% zygote settlement (Fig 5).

### Phlorotannin concentration

**Fig 6:**
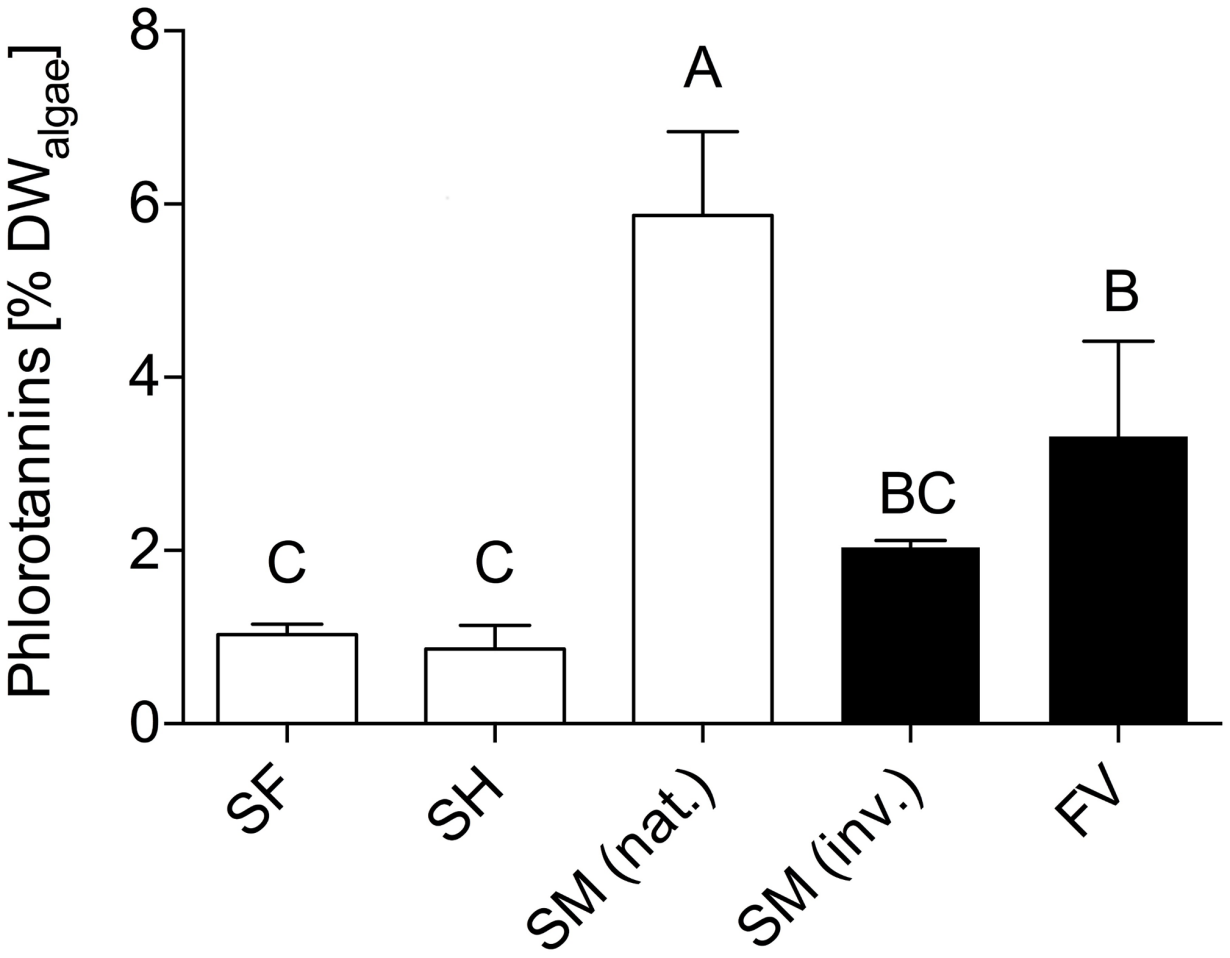
Algal phlorotannin content. Tissue phlorotannin content (means + SD; n = 3) in *Sargassum* spp. from Japan (white bars; *S*. *muticum* (*SM* (nat), *S*. *fusiforme* (*SF*), *S*. *horneri* (*SH*)) and *S*. *muticum* (*SM* (inv.) and *F*. *vesiculosus* (*FV*) from the North Sea (black bars). Different letters above the bars indicate significant differences between tissue concentrations (ANOVA, Tukey post hoc test, p < 0.05).

Mean phlorotannin concentration of *F*. *vesiculosus* and native *S*. *muticum* ranged from 3.32 to 5.87% of algal dry weight respectively and exceeded other *Sargassum* spp. by up to 6.7 times in the case of *S*. *horneri* (Fig 6).

## Discussion

The introduction of alien species can affect native community structure significantly [1, 49]. The secondary metabolites of introduced species may facilitate the colonization and range expansion of these alien species [7]. This study compares chemical activity of invasive and native algal species. We could demonstrate that extracts of *S*. *muticum* from Japan exhibited stronger fouling and settlement defence compared to the North Sea brown alga *F*. *vesiculosus* in most bioassays. Additionally, the amount of phlorotannins in *S*. *muticum* (nat.) tissue exceeded those in other *Sargassum* spp. and *F*. *vesiculosus*. All *Sargassum* spp. from Japan significantly inhibited bryozoan–and *F*. *vesiculosus* zygote settlement. Decreased antifouling defence of *S*. *muticum* (inv.) compared to native Japanese *Sargassum* species is consistent with our recent study comparing the chemical herbivore deterrence of the same species [50].

Antifouling compounds must either be present at the host’s surface or released into the surrounding water at a concentration that deters ecologically relevant fouling organisms [51]. Thus, surface extracts provide a more realistic estimation of the natural antifouling activity. An effective antifouling activity of the surface extracts is demonstrated by the higher antibacterial activity of *Sargassum* spp. surface extracts compared to crude extracts. However, not only surface extracts, but crude extracts at natural concentrations of all *Sargassum* spp., except *S*. *horneri*, caused significant diatom mortality. The variable activity of crude and surface extracts and the inconsistent antifouling effect of surface extracts among different algal species correlates with earlier findings [52]. This can be explained by the fact that the metabolites that act as antifoulants are stored within specialized structures inside the algal thallus and come to the surface in variable amounts [53]. The concentration of substances on algal surfaces is often less than inside its thallus [54], and sometimes even insufficient to inhibit fouling [55]. This might explain the high antifouling activity of crude extracts and the variable effect of surface extracts in our study. Interestingly, for some species like *S*. *horneri* and native *S*. *muticum* (nat.) higher concentrations of extracts lead to growth stimulation but not growth inhibition. This might be explained by the additional amount of nutrients in the higher concentrated extracts that override the growth inhibiting activities.

### Invading the North Sea: *Sargassum* spp. vs. *F*. *vesiculosus*

**Fig 7:**
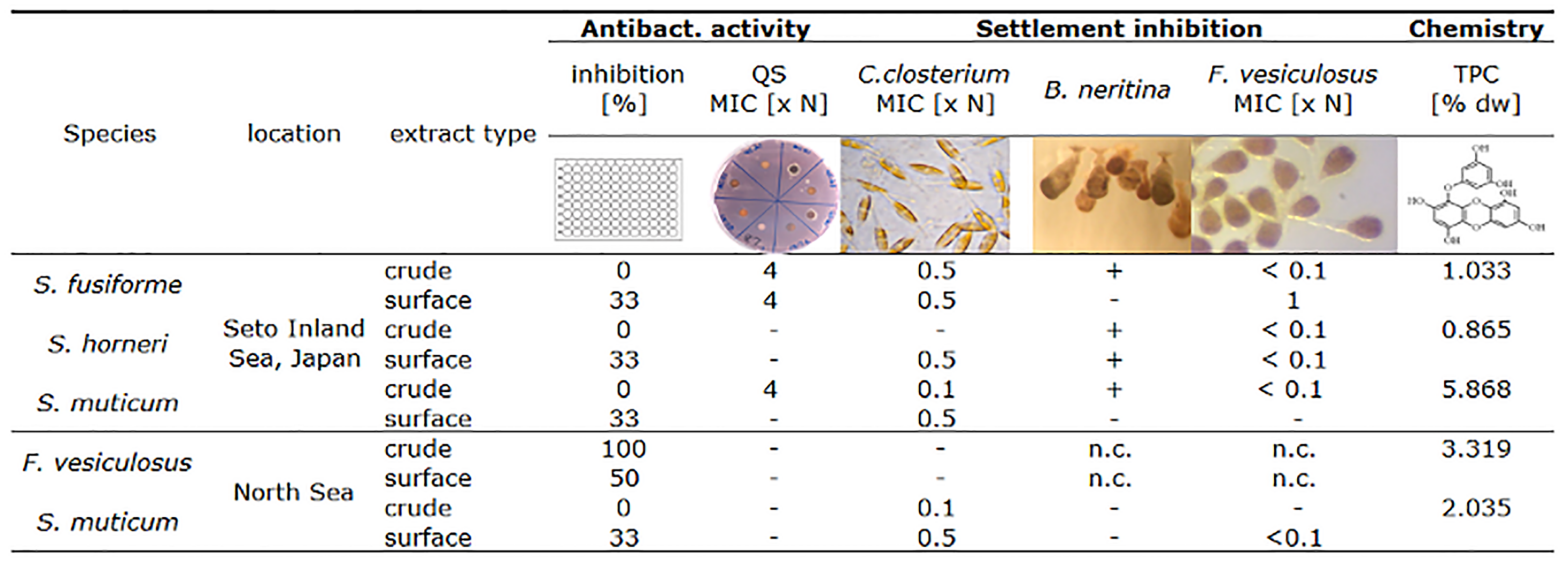
Summary of the results of the experiments comparing the chemical defence of the invasive and native *S*. *muticum*, *Sargassum* spp. from Japan, and the brown alga *F*. *vesiculosus* from the North Sea. The antibacterial activity is presented as the percent of inhibited bacterial strains and MIC-values are provided for inhibition of QS (X-times natural concentration, ‘-’ = no inhibition). The settlement inhibitory activity is presented as both, the MIC inhibiting diatom (*C*. *closterium*) growth and bryozoan (*B*. *neritina*) larval settlement (‘+’ = settlement inhibition after 24h; ‘-’ = no settlement inhibition; n.c. = not conducted). Algal zygote settlement inhibition is presented as the MIC inhibiting *F*. *vesiculosus* zygote settlement (as X-times natural concentration [x N]). The amount of phlorotannins in algal tissues is represented as the proportion of total phenolic content (TPC) per algal dry weight (dw).

*S*. *muticum* (nat.) was better defended against fouling and settlement compared to *F*. *vesiculosus* (Fig 7). This supports our hypothesis that invasion success is correlated with an effective chemical defence. Our results suggest a scenario where bioactive specimens from Japan were introduced into the North Sea facing the less defended competitor *F*. *vesiculosus*. The stronger defence properties of *S*. *muticum* might have facilitated its establishment and spread in the new habitat.

Native and invasive populations of *S*. *muticum* exhibited strongest antidiatom activity in comparison to surface extracts of *F*. *vesiculosus* which caused only a slight decrease of diatom densities. Antidiatom activity of *S*. *muticum* has been observed in previous studies and has been attributed to palmitic acid [56]. [57] found strong antibacterial activity of *F*. *vesiculosus* surface extracts, which could be partly attributed to associated diatoms. Thus, they suggested that harbouring a thin film of epiphytic diatoms might support the chemical defence of macroalgae against bacterial settlement. This assumption is supported by the strong antibacterial activity of *F*. *vesiculosus* in the present study. However, a layer of settled diatom can have different adverse effects. A study by [58] showed that herbivory and epibiosis were closely connected and the most fouled genotypes faced the largest grazing loss [58]. In fact, epibiosis decreased growth of *F*. *vesiculosus* in field and mesocosm experiments about 27% [58] and determined its depth distribution [59]. North Sea mesograzers preferred *F*. *vesiculosus* as food compared to *S*. *muticum* [50] and higher diatom densities on *F*. *vesiculosus* might further attract herbivores, thereby decreasing growth rate and fitness of *F*. *vesiculosus* and increasing the spreading potential of *S*. *muticum*.

In our study extracts of *Sargassum* spp. inhibited *Fucus* zygote settlement even at 0.1-times natural concentration. This might contribute to the invasion success of *S*. *muticum* in the North Sea as well. A few studies have demonstrated that seaweeds have the ability to suppress natural competitors through chemical agents [60–62]. Surprisingly, the decrease in zygote settlement was stronger when the extracts were diluted. To our knowledge this effect has not been described in the literature before and might be explained with additional nutrients supplied by the higher concentrated extracts.

*S*. *muticum* chemistry might prevent the settlement of early life stages of native species in adjacent areas, which could be regarded as a form of pre-emptive competition, a fundamental process in ecological communities [63]. Numerous studies have found that the *Sargassum* invasion is disturbance dependent [64–66], indicating that established undisturbed assemblages of native algae prevent invasion by *S*. *muticum*. The intertidal zone of Helgoland is characterized by strong wave action [67] and the wave induced removal of *F*. *vesiculosus* after strong storms might enable the colonization of *S*. *muticum* pioneer populations. Active metabolites of *S*. *muticum* causing settlement inhibition of *F*. *vesiculosus* zygotes and alteration of microbial biofilms might consequently contribute to the establishment and spread of *S*. *muticum* into the North Sea.

A strong antibacterial activity of *F*. *vesiculosus* was observed in this study. Similarly, the high antibacterial activity of *F*. *vesiculosus* has been reported previously [21, 27, 57, 68] and might be partly attributed to its perennial life strategy. Furthermore, the high bacterial densities in the North Sea (0.84 to 3.69 x 10^6^ cells ml^-1^; [69]) cause a high bacterial fouling pressure on *F*. *vesiculosus*. As a result of these tough life conditions, *F*. *vesiculosus* might have developed mechanisms to reduce the impacts of bacterial damage and maintain its physiological integrity during and throughout emersion [70]. In contrast, *S*. *muticum* has a pseudo-perennial life cycle [71]. Thus, *S*. *muticum* renews its tissues every year, thereby removing epibiotic organisms. This can explain a weaker antibacterial activity of *S*. *muticum* in comparison with *F*. *vesiculosus*. Additionally, the weak antibacterial activity of *Sargassum* spp. extracts might be the result of a species specific mode of action [72, 73]. *Sargassum* spp. might promote the growth of specific bacterial strains as indicated by the significant growth stimulation of certain bacteria on different *Sargassum* spp. crude extracts. A controlled bacterial community could have synergistic effects and might protect the algal surface from additional fouling organisms by releasing bacterial antifouling substances in the surrounding seawater [12, 74, 75]. [76] showed that common macroalgal metabolites, such as dimethylsulphopropionate (DMSP) and the amino acids proline and alanine inhibited surface attachment of some bacteria (e.g. *Cytophaga* sp.), while enhancing the attachment of others (e.g. *Rheinheimera baltica*). A study comparing antifouling properties of crude extracts from *S*. *muticum* and *Sargassum* spp. from Oman showed that *S*. *muticum* extract inhibited bacterial growth of four bacterial strains in the laboratory, while promoting bacterial settlement in the field [73]. These data support the concept that *S*. *muticum* metabolites enhancing the attachment of certain bacteria with antifouling properties and inhibiting attachment of others.

Extracts of *F*. *vesiculosus* showed no anti-QS activity in this study but extracts of *S*. *fusiforme* from Japan inhibited bacterial QS without any toxic effect. On contrary, high anti-QS activity of *Sargassum* species was observed in previous studies [17, 18, 35]. The low anti-QS activity found in this study might be due to differences in extraction techniques or alternatively differences in microbial communities associated with different *Sargassum* species, which could play a role in the anti-QS activity [17, 18].

#### Adaptation of chemical defence after invasion: *S*. *muticum* (nat.) vs. *S*. *muticum* (inv.)

Antibacterial and antidiatom activities of native and invasive *S*. *muticum* populations were similar. This might be due to similar chemical compounds in both algal populations. Additionally, similar bacterial densities were observed in the Seto Inland Sea (0.32 to 3.4 x 10^6^ cells ml^-1^; [77]) and the German North Sea (0.84 to 3.69 x 10^6^ cells ml^-1^; [69]), indicating similar bacterial fouling pressure. Both surface extracts inhibited the growth of the same test strains, revealing similar antibacterial defence and no evolutionary adaptation to prevent growth of bacteria in the new environment.

*S*. *muticum* (nat.) exhibited stronger anti-settlement activity against *B*. *neritina* larvae compared to its allopatric population from the North Sea. At the time *S*. *muticum* entered the North Sea, *B*. *neritina* was not a sympatric species, since it was first recorded in the German North Sea only in 2010 [24], approximately 30 years after *S*. *muticum* establishment. In contrast, *B*. *neritina* occurred around Japan at least since 1960 [78]. Thus, one could speculate that *S*. *muticum* (inv.) lost or reduced antifouling metabolites in the North Sea during the time period without fouling pressure of *B*. *neritina*. Phlorotannins have been shown to be highly effective against larval settlement [21] and the higher phlorotannin concentration of *S*. *muticum* (nat.) in our study compared to *S*. *muticum* (inv.) might contribute to its antifouling activity. Differences in phlorotannin concentration between genera depend on geographical location and there is a consensus among researchers that environmental factors have a significant impact on phenolic content of brown algae in general [79, 80].

*Fucus* zygote settlement was significantly reduced by native and invasive *S*. *muticum* crude extracts compared to the controls, even at 0.1-times natural concentration. This effect was again more pronounced with native *S*. *muticum* crude extracts. Native *S*. *muticum* populations in Japan experience stronger competition with other macroalgae since the algal biodiversity in Japan is much higher than in the North Sea (more than ten *Sargassum* species are competing among themselves in certain Japanese regions [81]. Consequently, a higher resource allocation to competitive traits might be an adaptive advantage and the reason for an increased bioactivity in Japanese *S*. *muticum* extracts. This is supported by results of our previous study on anti-herbivore defences of invasive and native *Sargassum* species [50]. Similar to this study, the native *Sargassum* spp. from Japan deterred feeding of dominant North Sea herbivores significantly more than the invasive *S*. *muticum* from the North Sea. Stronger anti-herbivore, antifouling and competitive properties of *S*. *muticum* (nat.) compared to invasive *S*. *muticum* represent an apparent paradox, which might be explained by the EICA hypothesis. It suggests that in regions of low competition pressure, selection will favour those genotypes with increased vegetative growth and/or reproductive efforts combined with reduced resource allocation to enemy defence. A plastic response to reduced enemy pressure, as stated by the PICA hypothesis, is equally conceivable and could lead to the same outcome of reduced defences as a trade-off to increase competitive ability. In fact, *S*. *muticum* is one of the smaller *Sargassum* species in Japan (75–120 cm; [82, 83]) but grows considerably larger when introduced in new areas and it reaches length of >4 m in the German North Sea [84].

#### Invasion success: *S*. *muticum* (nat.) vs. non-invasive relatives

While *S*. *muticum* (nat.) exhibited stronger antifouling activities in most assays, there is no clear evidence that it is better defended than its native, non- or weak invasive relatives. Despite significantly higher phlorotannin content in *S*. *muticum* (nat.), there is no indication of stronger antifouling defence, suggesting other metabolites can act as antifoulants. The antifouling activity of *S*. *horneri* has been assigned to chromanols, which are active against the mussel *Mytilus edulis*, the soft fouling macroalga *Ulva pertusa*, the diatom *Navicula annexa*, and the bacteria *Pseudomonas aeruginosa* [85]. Similar chemical antifouling and antiherbivore properties [50, 73] among native *Sargassum* spp. may suggest other traits besides or in conjunction with enemy defence, to be important for the establishment and dispersal of introduced species including tolerance to grazing [86, 87], faster growth or higher fecundity [87, 88], and a positive response to disturbance [89]. *S*. *muticum* has a high reproductive output, fast growth and great potential to colonize uninhabited areas [87, 89, 90]. It can be argued that the well-defended *S*. *fusiforme* and *S*. *horneri* might invade the North Sea in the future as well. *S*. *horneri* has recently been detected in California (USA), from where it spread rapidly along the southern region of the Pacific and might achieve higher growth rates with further dispersal [91]. *S*. *horneri* might continue its invasion into the North Sea as well. It might therefore be reasonable to include further *Sargassum* species in North Sea monitoring programs to prevent their establishment in European waters.

In conclusion, stronger chemical antifouling properties and competitiveness of *Sargassum* spp. compared to *F*. *vesiculosus* might contribute to the invasion success of *S*. *muticum* into the North Sea. The stronger chemical defence of *S*. *muticum* from Japan had helped this species to out compete *F*. *vesiculosus* by preventing the settlement of *F*. *vesiculosus* recruits and by altering the bacterial composition of biofilms. Stronger defence of native compared to invasive *S*. *muticum* might be the result of lower enemy pressure and competition in the North Sea compared to the Japan Sea leading to a resource allocation shift from the production of chemical defence metabolites to reproduction and growth as postulated by the EICA and PICA hypotheses. Since other tested *Sargassum* species from Japan are equally chemically defended like *S*. *muticum* from Japan, they might have potential to invade other ecosystems and should be included in other monitoring programs.

## Supporting information

S1 Fig Polarity of anti-QS compounds. Detection of the polarity of active compounds of those extracts exhibiting QS-inhibitory properties in previous assays (i.e. surface and crude extracts of *S*. *fusiforme* and crude extracts of native *S*. *muticum*) by thin layer chromatography (TLC) using the reporter strain *C*.*violaceum* CV017 on C18 reversed-phase TLC plates and a mixture of DCM: EtOAc: hydrochloric acid 10% solution (18:1:1) as mobile phase. Inhibition zones of agar overlays revealed that QS inhibitory compounds were unipolar.
(TIFF)

S2 Fig Morphological changes of *Cylindrotheca closterium* grown on different algal extracts. *Cylindrotheca closterium* growth after 7 d in the presence of tissue concentrated extracts of *S*. *fusiforme*, *S*. *horneri* and the solvent control.
(TIFF)

S1 Table Data of algal experiments. Results of (1) bacterial growth inhibition experiment, (2) diatom growth inhibition assay, (3) larval settlement inhibition assay, (4) Fucus zygote setltement inhibition assay, and (5) Phlorotannin [%DW] measurement of algal tissue.
(XLSX)
