# Supplementary figures and images for: The role of chemical antifouling defence in the invasion success of Sargassum muticum: A comparison of native and invasive brown algae
Source: PLoS One. 2017 Dec 21;12(12):e0189761. doi: 10.1371/journal.pone.0189761 (PMC5739409; doi:10.1371/journal.pone.0189761)

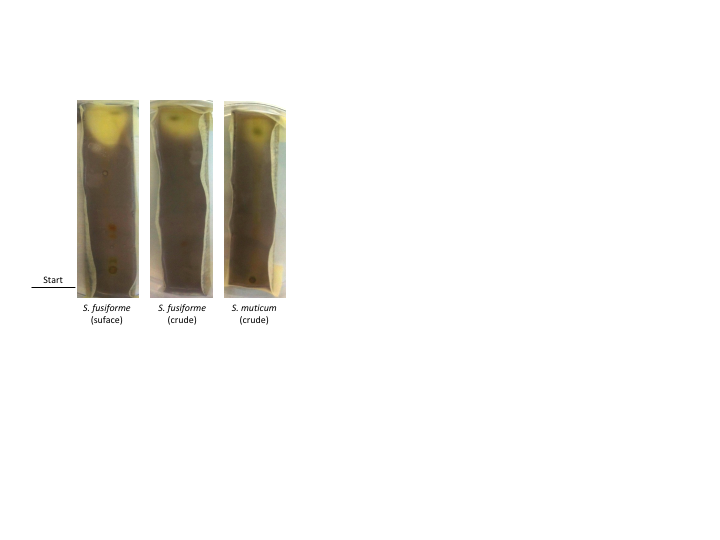

Supplement: S1 Fig — Detection of the polarity of active compounds of those extracts exhibiting QS-inhibitory properties in previous assays (i.e. surface and crude extracts of S. fusiforme and crude extracts of native S. muticum) by thin layer chromatography (TLC) using the reporter strain C.violaceum CV017 on C18 reversed-phase TLC plates and a mixture of DCM: EtOAc: hydrochloric acid 10% solution (18:1:1) as mobile phase. Inhibition zones of agar overlays revealed that QS inhibitory compounds were unipolar. (TIFF) [file pone.0189761.s001.tiff]

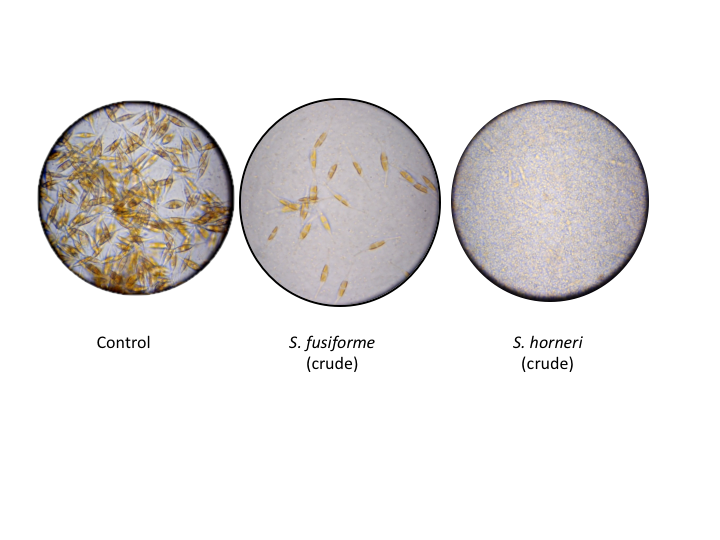

Supplement: S2 Fig — Cylindrotheca closterium growth after 7 d in the presence of tissue concentrated extracts of S. fusiforme, S. horneri and the solvent control. (TIFF) [file pone.0189761.s002.tiff]
